# Microwave-Assisted Acid Hydrolysis vs. Conventional Hydrolysis to Produce Sapogenin-Rich Products from Fenugreek Extracts

**DOI:** 10.3390/foods11131934

**Published:** 2022-06-29

**Authors:** Joaquin Navarro del Hierro, Emma Cantero-Bahillo, M. Teresa Fernández-Felipe, Diana Martin

**Affiliations:** 1 Departamento de Producción y Caracterización de Nuevos Alimentos, Instituto de Investigación en Ciencias de la Alimentación (CIAL) (CSIC–UAM), 28049 Madrid, Spain; joaquin.navarrodel@uam.es (J.N.d.H.); emma.cantero@uam.es (E.C.-B.); mariateresa.fernandezf@uam.es (M.T.F.-F.); 2 Sección Departamental de Ciencias de la Alimentación, Facultad de Ciencias, Universidad Autónoma de Madrid, 28049 Madrid, Spain

**Keywords:** saponin, aglycone, MAAH, time, temperature, diosgenin, bioactivity, antioxidant, DPPH, lipase inhibition

## Abstract

The acid hydrolysis of saponins is commonly performed by conventional heating to produce sapogenin-rich products of bioactive interest, but alternative hydrolysis methods and their impact on bioactivity have been unexplored. We compared the conventional method with microwave-assisted acid hydrolysis (MAAH) of a commercial saponin-rich extract from a typical saponin source, fenugreek, focusing on the study of temperature (100, 120, 130, 140, 150 °C) and time (10, 20, 30, 40 min) of hydrolysis. The impact of these factors was assayed on both the sapogenin yield and the bioactivity of the hydrolyzed products, specifically their antioxidant and lipase inhibitory activities. The highest sapogenin content (34 g/100 g extract) was achieved by MAAH at 140 °C and 30 min, which was higher than conventional hydrolysis at both reference conditions (100 °C, 60 min, 24.6 g/100 g extract) and comparative conditions (140 °C, 30 min, 17 g/100 g extract) (*p* < 0.001). Typical steroid artifacts from sapogenins were observed in very small amounts, regardless of the method of hydrolysis. Antioxidant activity of MAAH hydrolyzed extracts (around 80% DPPH inhibition) was barely affected by time and temperature, but pancreatic lipase inhibitory activity was higher (>65%) at lower MAAH temperature (<130 °C) and time (<30 min) of hydrolysis. MAAH is shown as a valid alternative to produce selective sapogenin-rich extracts from fenugreek with minor impact on their bioactivities, and whose magnitude can be modulated by the hydrolysis conditions.

## 1. Introduction

Saponins are naturally occurring glycosides widely reported in many families of edible seeds and plants (*Fabaceae*, *Liliaceae*, *Caryophyllaceae*, *Agavaceae*, among others), and include a diversified group of compounds whose structure contains one or more sugar chains linked to a steroidal or triterpenoid sapogenin, of hydrophobic nature [1,2]. Saponins have been heavily studied in the past and recently due to the numerous evidence-based works demonstrating their hypolipidemic, anti-inflammatory, antibacterial and anticancer activities, among others, either alone or when contained in natural extracts [3,4,5,6]. However, the hydrophobic part or sapogenin has been shown to exert certain stronger bioactivities than native saponin, due to more favorable chemical properties [7]. In this sense, the edible seed fenugreek (*Trigonella foenum-graecum*), with multiple proven health benefits, is one of the most known sources of steroid saponins, diosgenin being one of its major sapogenins [8]. This aglycone has been widely studied in terms of its effects on hypolipidemia, cancer and inflammation, among others [9,10,11]. For example, in terms of the potential management of blood glucose levels, diosgenin has demonstrated very recently to exhibit a stronger inhibition towards dipeptidyl peptidase-IV (DPP-IV) enzyme than its precursor saponin protodioscin, although this last compound had a slightly stronger inhibitory activity towards α-amylase and α-glucosidase [12]. In silico, diosgenin and yamogenin displayed stronger binding abilities than protodioscin to estrogen receptor α (ERα), key in hormone-dependent and independent tumors in which the activation of ERα is linked to cancer progression [13]. Gupta et al. [14] showed that a diosgenin enriched *Paris polyphylla* rhizome extract demonstrated a potent in vitro antioxidant effect by effectively quenching 2,2-diphenyl-1-picrylhydrazyl (DPPH), nitric oxide dismutase (NOD), superoxide dismutase (SOD) and reducing power free radicals. In fenugreek, diosgenin-rich extracts obtained after acid hydrolysis of saponin-rich products have also been demonstrated very recently to maintain strong in vitro lipase-inhibitory activities [15].

However, fenugreek or most sources of edible seeds do not contain free sapogenins or the existent concentrations in the raw material are minimal. Hence, for the specific obtention of sapogenin-rich extracts from these matrixes, an additional step needs to be performed once the saponin-rich extracts are obtained. It fundamentally implies the release of the aglycone part from the sugar moieties, and can be performed chemically (acid or alkaline), enzymatically, by microbial methods and even by subcritical water, leading to the formation of sapogenins, prosapogenins, sugar residues, or monosaccharides depending on the hydrolysis method and conditions performed [16]. In general, it seems that enzymatic methods are less effective than chemical hydrolysis, making the acid hydrolysis of saponins the preferred procedure, which has been more extensively used and explored. It is important to consider that during the chemical hydrolysis of saponins, specific conditions, such as temperature, time of reaction, and type of chemical catalyst could affect the stability of saponins, the yield of final products, and the generation of artifacts [17,18,19,20]. Concerning chemical catalysts, the common acid employed during acid hydrolysis is hydrochloric acid [21,22,23]. Concerning the time of hydrolysis, 2–3 h of reaction time are generally applied, but longer hydrolysis times have also been found, which may cause the degradation of the released sapogenins at the expense of unnecessary energy consumption. Additionally, prolonged heating times during acid hydrolysis of saponins can cause artifact formation, low yields and low selectivity [20,24]. In this regard, we have recently optimized the hydrolysis time of saponins from fenugreek and other edible seeds by a conventional heating method, establishing 1 h as the most optimal time in which the highest yield of sapogenins was obtained, longer times lead to reduced sapogenin contents and degradations [19].

Concerning other alternative methods to conventional heating for the hydrolysis of compounds in general, microwaves have been employed over the last few decades as a novel, more efficient and greener method for the hydrolysis of different molecules, such as cellulose, seed gums, chitosan, proteins and olive oil polyphenols, among many others [25,26,27,28,29,30]. However, the potential of microwave-assisted hydrolysis has not been fully exploited when considering its use for the hydrolysis of saponins from natural sources as an alternative method to conventional thermal methods. For example, Wang et al. [31] studied the most optimal microwave conditions (temperature, time, concentration of solvent and ratio of solvent to solid) for the production of diosgenin from yam. These authors were able to produce, after 20 min of microwave irradiation, a very similar amount of diosgenin to the amount obtained in conventional heating after 5 h. However, similar studies assessing microwave hydrolysis conditions on other different saponin-rich natural sources, such as fenugreek, are notably scarce. More recently, Colson et al. [32] evaluated how the alkali microwave hydrolysis of quinoa saponins at pHs ranging from 7 to 14 and temperatures ranging from 60 to 180 °C affected the composition and hemolytic activity of the final hydrolysates. In this specific regard, it would be very interesting to consider how the different conditions assayed during hydrolysis may modulate the subsequent bioactivity of the resulting sapogenin-rich extracts in order to selectively produce those extracts that satisfy the specific needs. In other words, a specific hydrolysis condition might lead to the richest extracts in terms of the sapogenin content whilst not to the strongest extracts in terms of their bioactivity, due to the potential impact of the hydrolysis conditions on the bioactivity of other co-existing compounds. On the opposite side, extracts with the strongest bioactivity might not have the richest sapogenin content, but such content might be enough for the specific needs. Therefore, the simultaneous consideration of the impact of hydrolysis conditions on both the yield of the target bioactive molecule and the bioactivity of the hydrolyzed products might be of interest.

The aim of this work was to evaluate the use of microwave-assisted acid hydrolysis (MAAH) as an alternative technology to conventional heating to produce sapogenin-rich extracts by acid hydrolysis from fenugreek extracts. To perform this study, a commercially available saponin-rich fenugreek extract was used. Two sequential trials were performed, a first one to study the effect of the temperature of hydrolysis on the sapogenin yield, and a second one to study the effect of the hydrolysis time. The generation of sapogenin artifacts was also considered in both studies. Finally, in order to study how these two factors of hydrolysis could modulate the bioactivity of the extracts produced under the different conditions, two popular bioactivities of natural extracts were monitored, as the pancreatic lipase inhibitory activity and the antioxidant activity.

## 2. Materials and Methods

### 2.1. Reagents and Materials

A commercial saponin-rich extract from fenugreek (“fenugreek extract”, Fenfuro™) was purchased from Chereso (Panchkula, India). The saponin content of the extract was determined to be 82% by high-performance liquid chromatography-diode array detection (HPLC-DAD), as described by Navarro del Hierro et al. [33].

Hydrochloric acid (2 M) was from Merck KGaA (Darmstadt, Germany). Ethyl acetate and methanol were from Macron (Gliwice, Poland). Trizma base, maleic acid, sodium chloride, calcium chloride, phosphatidyl choline from egg yolk, lipase from porcine pancreas, 4-methylumbelliferyl oleate (4-MUO), diosgenin and 2,2-diphenyl-1-picrylhydrazyl (DPPH) were from Sigma-Aldrich Chemie GmbH (Steinheim, Germany).

### 2.2. Production of Sapogenin-Rich Extracts by Conventional Thermal Acid Hydrolysis

The hydrolysis of the fenugreek extract by conventional thermal hydrolysis was performed according to Navarro del Hierro et al. [33]. The extract was solubilized in HCl 2 M at a ratio of sample to acid solution of 1:50 (*w*/*v*). The mixture was heated at 100 °C (Stuart™ block heater, Cole-Parmer, Staffordshire, UK) at different times for comparative purposes with MAAH. Hydrolyzed samples were ice-cooled for 10 min, extracted with ethyl acetate at a ratio of 1:1 (*v*/*v*) by vortex agitation for 1 min and further centrifuged for 5 min at 3400× *g*. The upper phase was collected and the lower phase was extracted again with ethyl acetate under the same conditions. Both collected phases were combined, evaporated until dryness and the obtained hydrolyzed extract was stored at −20 °C until further use. This procedure was performed in duplicate and the hydrolysis extraction yield was calculated as follows:

Yield (%) = (weight of hydrolyzed extract/weight of initial fenugreek extract) × 100

A control non-hydrolyzed fenugreek extract was also produced by extracting the commercial fenugreek extract with ethyl acetate under the same described conditions, although solubilizing it in water instead of acid solution.

### 2.3. Production of Sapogenin-Rich Extracts by Microwave-Assisted Acid Hydrolysis (MAAH)

The hydrolysis of fenugreek extract by MAAH was performed as follows. The extract was solubilized in HCl 2 M at a ratio of sample to acid solution of 1:50 (*w*/*v*). The mixture was poured into a high pressure vessel and built into its own complete segment from an MLS 1200 Mega microwave system (Milestone Srl, Sorisole, Italy). Two sequential trials were performed. A first trial tested different temperatures (100, 120, 130, 140 and 150 °C) at constant time of hydrolysis (30 min), and a second one tested different processing times (10, 20, 30 and 40 min) at a constant temperature of hydrolysis (140 °C, selected according to the first trial). The theoretical power output of the equipment was 800 W and the real power output of the magnetron was indirectly determined by measuring the increase in temperature of the water after microwave irradiation, according to Bizzi et al. [34]. The real power delivered inside the cavity was 752 W. The temperature of the samples during the different reaction conditions was measured with a fiber optic temperature sensor directly from a control high-pressure vessel containing distilled water. Once the reaction was finished, the complete segment was directly placed into an ice bath for 20 min and then unsealed for the extraction of the hydrolyzed extract by ethyl acetate. This step was performed exactly as described in Section 2.2. The procedure was performed in duplicate and the hydrolysis extraction yield was calculated as previously explained.

### 2.4. Analysis of the Sapogenin-Rich Extracts by GC-MS

The sapogenin content of all the extracts was characterized and quantitated in an Agilent 7890A GC-MS-FID (Agilent Technologies, Santa Clara, CA, USA), with previous derivatization of the samples with BSTFA at 10 mg/mL (60 min, 75 °C) [19]. The equipment comprised a split/splitless injector, G4513A autoinjector, an electronic pressure control, and a 5975C triple-axis mass spectrometer detector. The column employed was an Agilent HP-5MS capillary column (30 m × 0.25 mm i.d., 0.25 µm phase thickness) and the carrier gas was helium at a flow of 2 mL/min. Sample injections (1 µL) were conducted in splitless mode. The injector temperature was 260 °C and the mass spectrometer ion source and interface temperatures were 230 and 280 °C, respectively. The temperature of the oven was initiated at 50 °C, held for 3 min, and increased at a rate of 15 °C/min to 310 °C, which was then held for 25 min. The mass spectra were obtained by electron ionization at 70 eV. The scanning speed was 1.6 scans/s in a mass range of m/z 30–700. Identification of compounds was performed by the NIST MS Data library and by the mass spectra according to the literature [19,33]. Quantification of sapogenins was performed under the same conditions with a calibration curve from the commercial standard diosgenin as the most abundant sapogenin from fenugreek, which was derivatized following the same procedure as the samples. The total sapogenin content of the commercial fenugreek extract (“fenugreek extract”) was determined to be 0.37%.

### 2.5. Pancreatic Lipase Inhibition Assay

The inhibitory activity against pancreatic lipase of all the different extracts was assayed by using 4-MUO as substrate, according to Herrera et al. [35]. The assay was performed under simulated intestinal conditions by using a digestion buffer (Trizma–Maleic 100 mM pH 7.5, 0.15 M NaCl, and 5.1 mM CaCl_2_) with bile salts (7.81 mg/mL) and lecithin (3.12 mg/mL). The reaction mixture consisted of 0.5 mL of extract solution in digestion buffer at 3 mg/mL, 0.5 mL of freshly-prepared pancreatic lipase at 1 mg/mL (0.01 g of lipase in 10 mL digestion buffer, stirred for 10 min and centrifuged for 10 min at 2688× *g*), and 1 mL of 4-MUO solution at 0.1 mM in digestion buffer. The final concentration of the extracts in the reaction mixture was 0.75 mg/mL. Control samples were prepared following the same procedure in absence of extracts, and extract controls were prepared in absence of lipase and 4-MUO. The reaction mixture was placed in an orbital incubator (Titramax 1000, Heidolph Instruments, Schwabach, Germany) at 37 °C with shaking (250 rpm) for 20 min and protected from light. After incubation, three aliquots of 150 µL were added to a 96-well plate. The amount of 4-MUO hydrolyzed by lipase in presence of the extracts was measured using an Infinite M200 fluorescence microplate reader (Tecan, Salzburg, Austria) at an excitation wavelength of 350 ± 10 nm and an emission wavelength of 450 nm. The inhibition of the pancreatic lipase by the extracts was calculated by using the following formula:

% Inhibition = 100 − [(F_extract sample_ − F_extract control_)/F_control sample_] × 100

where F_extract sample_ is the fluorescence of the reaction with extract, F_extract control_ is the fluorescence of the extract controls in absence of enzyme and substrate, and F_control sample_ is the fluorescence of the reaction mixture without extract.

### 2.6. Antioxidant Activity Assay

The antioxidant activity of all the different extracts was studied by the DPPH radical scavenging assay according to Blois [36]; 40 µL of the extract solution in methanol at 5 mg/mL were mixed with 560 µL of a DPPH solution in methanol (0.06 mM). The final concentration of the extracts in the reaction mixture was 0.3 mg/mL. Samples were incubated for 60 min in darkness at room temperature. Control samples were prepared in absence of extracts, following the same procedure. Absorbance was measured at 517 nm and methanol was used as a blank. Determinations were made in triplicate and the antioxidant activity was expressed as the percentage of DPPH inhibited according to the following formula:

% DPPH Inhibition = 100 − (Absorbance_sample_/Absorbance_control_) × 100

### 2.7. Statistical Analysis

Statistical analyses were performed by means of the general linear model procedure of the SPSS 26.0 statistical package (SPSS Inc., Chicago, IL, USA) by one-way analysis of variance. Differences were considered significant at *p* ≤ 0.05. Post-hoc Tukey’s tests were performed in order to establish significant differences. Pearson correlation tests were conducted for additional analyses.

## 3. Results and Discussion

### 3.1. Hydrolysis Extraction Yield and Sapogenin Content of Hydrolyzed Extracts

#### 3.1.1. Effect of MAAH Temperature

**Figure 1:**
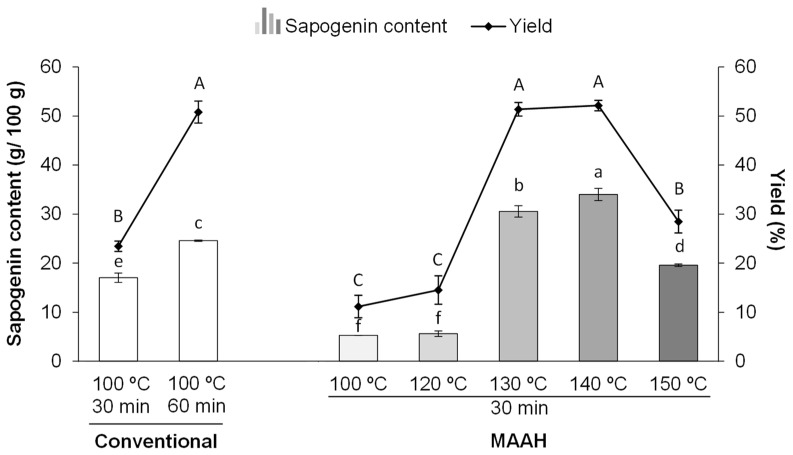
Sapogenin content (g/100 g) and hydrolysis extraction yield (%) of sapogenin-rich extracts obtained by conventional hydrolysis and microwave-assisted acid hydrolysis (MAAH) at different temperatures. Standard deviations are indicated by error bars. Mean values with different letters (A–C, yield; a–f, sapogenin content) are significantly different (*p* ≤ 0.05).

Considering the importance of temperature as a key parameter for the hydrolysis of saponins, a first trial was performed to assess the effect of microwave temperature and its comparison with conventional heating. Initially, conventional hydrolysis at 100 °C and 60 min was established as the reference conditions according to previous studies in which the optimal time of hydrolysis of saponin-rich seed extracts (fenugreek among them) was confirmed to be 60 min under this same method [19]. As shown in Figure 1, the hydrolysis extraction yield and sapogenin content of extracts obtained under the reference conditions were 50.8% and 24.6 g/100 g, respectively. Nevertheless, shorter times of 30 min at 100 °C were also assayed under conventional heating (Figure 1) for comparative purposes with MAAH, taking into consideration the generally assumed shorter times of heating used during microwave irradiation [37].

Taking these values of conventional heating as a reference, the results of MAAH at different temperatures were compared in Figure 1. The hydrolyzed fenugreek extract produced under MAAH at 100 °C and 30 min had an 11.2% extraction yield and 5.2 g of sapogenins per 100 g of extract, with both values being significantly lower than those described in both conventional conditions (100 °C, 30 min and 100 °C, 60 min) (*p* < 0.001). It must be noted that the sapogenin content of the commercial fenugreek extract used in this study was also analyzed in order to know the initial value of sapogenins of the sample before hydrolysis and after extraction with ethyl acetate (extraction yield = 7.1%), and it was determined that it contained 5.3 g of sapogenins per 100 g of ethyl acetate extract. Thus, considering the slightly higher hydrolysis extraction yield (11.1%) of the hydrolyzed MAAH extract at 100 °C for 30 min and the similar sapogenin content (5.2 g of sapogenins/100 g of extract), only a very subtle hydrolysis effect was observed when compared to the non-hydrolyzed commercial fenugreek extract. Similarly, at 120 °C of MAAH, there were no significant differences compared to 100 °C and thus it seems barely noticeable that hydrolysis was taking place.

Nevertheless, when the temperature was increased from 120 to 130 °C and 140 °C, both the hydrolysis extraction yield and sapogenin content increased dramatically. Thus, the highest sapogenin content of the hydrolyzed extracts was found at 140 °C (34 g/100 g), although there were no differences in terms of the extraction yield at either 130 or 140 °C (around 50% yield). It is interesting to remark that the sapogenin content in these extracts obtained with MAAH for 30 min was higher than in conventional heating for 60 min and 30 min. Additionally, it must be highlighted that temperatures above 140 °C during MAAH clearly led to a reduced hydrolysis extraction yield and sapogenin content, as observed at 150 °C (Figure 1). The sapogenin distribution of the hydrolyzed extracts produced at different MAAH temperatures is shown in Table S1.

The effect of temperature on the microwave hydrolysis of saponins has been very scarcely studied in the literature. Wang et al. [31] explored five different temperatures (80 °C, 90 °C, 100 °C, 110 °C, and 120 °C) during the microwave irradiation of saponin extracts from *Dioscorea zingiberensis*, a well-known natural source of diosgenin-containing saponins. The optimal temperature of hydrolysis was found to be 100 °C, as higher temperatures were related to the instability of diosgenin and the possible destruction of its structure. The lower optimal temperature found by these authors could be explained by the use of acid-functionalized ionic liquids instead of conventional solvents, such as hydrochloric acid, as ionic liquids contain a pair of ions and have a high density of strong dipoles, making them suitable for microwave absorption [38]. Besides, the introduction of acid functional groups has been related to high acid density, adjustable acidity, uniform acid intensity distribution and durable acidity [31]. However, other studies focusing on the MAAH of saponins or fenugreek extracts using conventional acids as HCl have not been described. As a summary, the current study shows that temperatures above 120 °C seem to be necessary for an efficient MAAH of fenugreek saponins, but temperatures above 140 °C might negatively impact the stability of the target products.

Additionally, in all cases it could be clearly observed that the hydrolysis extraction yields strongly correlated with the sapogenin content of the extracts regardless of the hydrolysis condition (r = 0.95, *p* < 0.001) (Figure 1). This would suggest that the extraction yield was widely modulated by the release of sapogenins during hydrolysis and the rest of the compounds contained in the extract did not affect the yield and release of sapogenins in a very determinant manner. This outcome might be interesting for a preliminary prediction of the approximate sapogenin content of these kinds of extracts by considering *a priori* the hydrolysis extraction yield.

**Figure 2:**
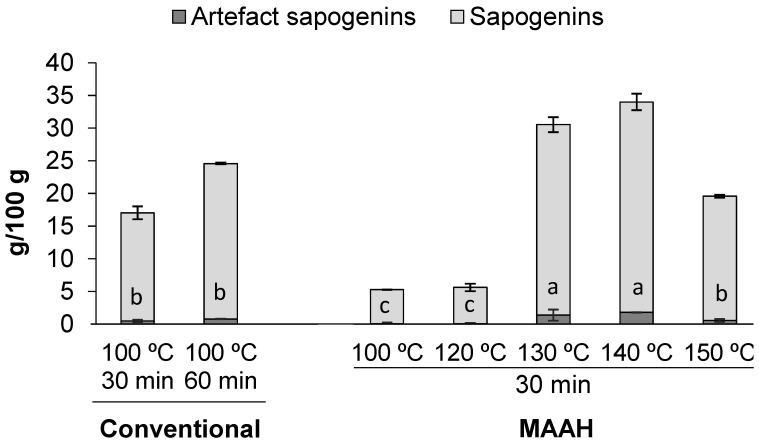
Sapogenin and artifact content (g/100 g) of sapogenin-rich extracts obtained by conventional hydrolysis and microwave-assisted acid hydrolysis (MAAH) at different temperatures. Standard deviations are indicated by error bars. Mean values with different letters (a–c) are significantly different (*p* ≤ 0.05).

As previously explained, the production of artifacts under hydrolysis conditions should be taken into account for the specific case of saponins [39]. The analysis of the extracts by GC-MS-FID allowed for the detection of the presence of some of these typically described artifacts among the steroid sapogenins found in fenugreek (Figure 2). These compounds have been described to originate from the dehydration of the C-3 hydroxyl group of diosgenin and yamogenin to give a major pair of 25R and 25S spirosta-3,5-dienes of 396 molecular mass by GC-MS, as well as another minor pair of isomeric dienes of unknown structure [39,40,41]. As shown in Figure 2, the artifact content in all the samples was very low and it increased proportionally with the total sapogenin content of each extract. Remarkably, it was observed that temperature during MAAH did not seem to be a relevant factor in the production of artifacts, as confirmed at 150 °C, in which the artifact content diminished proportionally with the decrease of the total sapogenin content. The available information about the hydrolysis conditions that determine the formation of artifacts from saponins is really scarce. It was described that the use of sulfuric acid in 70% 2-propanol, instead of hydrochloric acid, seems to reduce the formation of these artifacts [40]. Greener acids might be worth to be explored during the MAAH of steroid saponins in order to possibly avoid the formation of these dienes, although the relevance of these artifacts, apart from reducing the purity of diosgenin in the resulting extracts, has not been sufficiently well documented.

#### 3.1.2. Effect of MAAH Time

The impact of the time of reaction should be considered when the hydrolysis of saponins is performed, due to the potential degradation of the released sapogenins at the expense of unnecessary energy consumption in the case of long hydrolysis times. Additionally, it has been shown that prolonged heating times during acid hydrolysis of saponins can cause artifact formation, low yields and low selectivity, but the available information about these effects due to the time of microwave heating of saponins is very scarce. Therefore, in a second trial, the study of the time of MAAH hydrolysis was approached. Based on the first assay, 140 °C was kept constant to explore different times of microwave hydrolysis. For comparative purposes, the effect of time on the hydrolysis of saponins was also performed under conventional heating at 140 °C.

**Figure 3:**
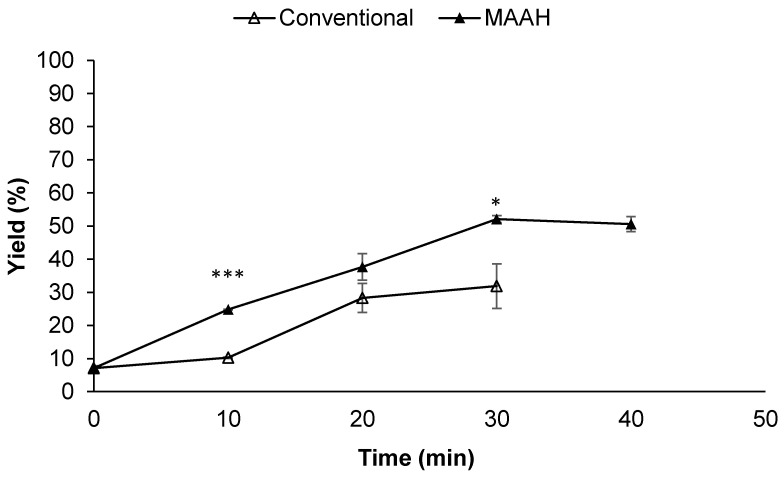
Hydrolysis extraction yield (%) of sapogenin-rich extracts from fenugreek obtained at different reaction times by conventional hydrolysis and microwave-assisted acid hydrolysis (MAAH) at 140 °C. Standard deviations are indicated by error bars. Mean values at the same time of hydrolysis are significantly different if *p* ≤ 0.05 (*) or *p* < 0.001 (***).

Focusing first on the extraction yield (Figure 3) a significantly higher yield was found for MAAH compared to conventional heating, regardless of the time of hydrolysis (*p* ≤ 0.001). The most noticeable difference between both methods was detected at 10 min of hydrolysis (*p* ≤ 0.05), with values of 10.3% for conventional hydrolysis and a value of 24.8% for microwave irradiation. In both cases, the hydrolysis extraction yield increased along with the reaction time until it reached 30 min, obtaining a yield of 52.1% by MAAH and 31.9% by conventional heating, being significantly different for both methods (*p* = 0.05). In the specific case of MAAH, longer times (40 min) did not lead to a performance improvement in terms of the extraction yield. This plateau was reached at 20 min for conventional heating.

**Figure 4:**
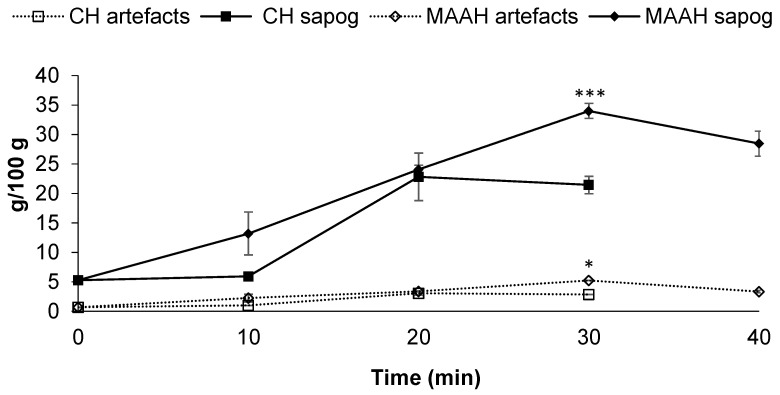
Sapogenin and artefact content (g/100 g) of sapogenin-rich extracts from fenugreek obtained at different reaction times by conventional hydrolysis and microwave-assisted acid hydrolysis (MAAH) at 140 °C. Standard deviations are indicated by error bars. Mean values at the same time of hydrolysis are significantly different if *p* ≤ 0.05 (*) or *p* < 0.001 (***).

Similar to the extraction yield, significantly higher sapogenin contents of the extracts were found for MAAH when compared to conventional heating (*p* ≤ 0.05), regardless of the time of hydrolysis (Figure 4). Sapogenin content in both cases increased, along with the hydrolysis time, until it reached 30 min for MAAH or 20 min for conventional heating, times at which the maximum sapogenin contents of each extract were confirmed, respectively. Thus, for MAAH, such a maximum value was 34 g/100 g, while those extracts produced under conventional heating after 20 min contained significantly fewer sapogenins (22.8 g/100 g) (*p* ≤ 0.001). Longer times of MAAH (40 min) led to a decrease in the sapogenin content (28.4 g/100 g). The sapogenin distribution of the hydrolyzed extracts produced at different MAAH times is shown in Table S2.

Wang et al. [31] also carried out a series of reaction time experiments, though shorter (5, 10, 15, 20 and 25 min), during the microwave hydrolysis of *Dioscorea zingiberensis* with acid-functionalized ionic liquids. They concluded that the optimal hydrolysis time under the conditions studied was 20 min, as an increase in the reaction time led to a slight decrease in the diosgenin yield. We have previously demonstrated that times above 1 h during conventional hydrolysis at 100 °C caused the degradation of sapogenins from different saponin extracts in the reaction medium [19]. Similarly, MAAH would cause the degradation of fenugreek sapogenins at times above 30 min and 140 °C, as the rate of sapogenin degradation may be overcoming the rate of saponin hydrolysis/sapogenin release.

Concerning artifacts, their content was very low in all extracts from both methods and affected by the hydrolysis time (Figure 4); such content was proportional to the sapogenin content of each extract, that is, the higher the total sapogenin content, the higher the artifact content, regardless of the hydrolysis method.

Additionally, as a complementary study, the effect of three HCl molarities (1, 1.5 and 2 M) was also evaluated on the hydrolysis extraction yield and total sapogenin content under the best conditions of MAAH just described (140 °C and 30 min) (Figure S1, Supplementary Materials). Both the yield and sapogenin content were lower when the MAAH was performed at 1 M compared to molarities of 1.5 and 2 M (*p* ≤ 0.05). Thus, HCl molarities above 1 M would be preferred for the production of the richest extracts in terms of their sapogenin contents, although a molarity under 1 M might be of interest in order to produce extracts with a lower environmental impact at the expense of a lower richness in sapogenins.

### 3.2. Effect of Acid Hydrolysis Conditions on the Bioactivity of Sapogenin-Rich Extracts

In order to evaluate how the different conditions of acid hydrolysis assayed during MAAH and conventional hydrolysis could impact the potential bioactivity of the resulting extracts, we studied their ability to inhibit the pancreatic lipase enzyme and their antioxidant activity by the DPPH radical scavenging assay. These two popular methods were only employed as tools to monitor the changing of already proven bioactivities for these types of extracts, due to either sapogenins or co-extracted compounds and the effects of different temperatures and time conditions of acid hydrolysis, by both MAAH and conventional hydrolysis.

#### 3.2.1. Effect on Inhibitory Activity against Pancreatic Lipase

The inhibition of this enzyme in the small intestine is of key interest for blocking the absorption of lipids from the diet and preventing excessive body weight gain [42]. In this regard, the inhibition of the pancreatic lipase by fenugreek and its derived bioactive compounds, including saponins, either isolated or contained in extracts has been well described both in vitro and in vivo [35,43,44,45,46,47,48].

**Figure 5:**
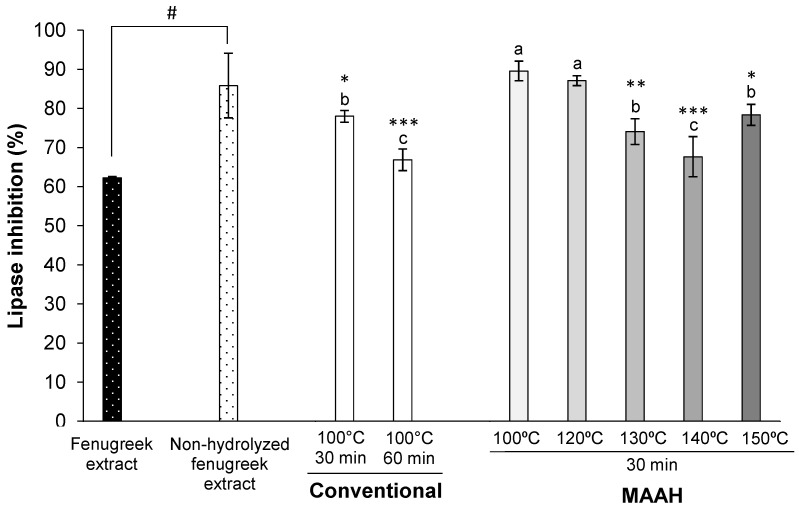
Effect of temperature on the pancreatic lipase inhibition (%) of fenugreek extracts and sapogenin-rich extracts obtained by conventional hydrolysis and microwave-assisted acid hydrolysis (MAAH). Standard deviations are indicated by error bars. Mean values with different letters are significantly different (*p* ≤ 0.05) and mean values are significantly different from the non-hydrolyzed fenugreek extract if *p* ≤ 0.05 (*), *p* ≤ 0.01 (**) and *p* ≤ 0.001 (***). Non-hydrolyzed fenugreek extract is different from fenugreek extract (#, *p* ≤ 0.001). “Fenugreek extract” refers to the commercial saponin mixture as received from the supplier. “Non-hydrolyzed fenugreek extract” refers to the “fenugreek extract” extracted with ethyl acetate at time 0 of hydrolysis.

We initially assessed the lipase inhibitory activity of the used commercial fenugreek extract and confirmed that it exhibited a 62% lipase inhibitory activity (Figure 5). Additionally, we assessed the lipase inhibitory activity of this same extract at time 0 of hydrolysis (non-hydrolyzed fenugreek extract extracted with ethyl acetate). Unexpectedly, the lipase inhibitory activity of the non-hydrolyzed fenugreek extract significantly improved (*p* < 0.001) to nearly 86% of inhibition when compared to the intact fenugreek extract (Figure 5). Given the lack of hydrolysis at time 0, we believe that the improvement in the inhibitory activity of this extract might be mainly related to the concentration of certain compounds due to the use of ethyl acetate as solvent, which are different from saponins or sapogenins and have the ability to inhibit the pancreatic lipase. This value was used as a reference for the study of the impact of the different hydrolysis conditions on the lipase inhibitory activity of the sapogenin-rich extracts obtained.

All the hydrolyzed extracts showed a great lipase inhibitory activity, above 65% in all cases (Figure 5). The lipase inhibitory activity of MAAH extracts at the lowest temperatures (100 and 120 °C) did not change with respect to the reference non-hydrolyzed fenugreek extract. However, higher temperatures (>120 °C) caused a lower inhibitory activity than the non-hydrolyzed fenugreek extract at time 0 of hydrolysis. Nevertheless, it should be remarked that, despite this negative effect, the inhibitory activity of such extracts was still very high (between 67 and 78%). We hypothesize that this lower inhibitory activity could be related to a “diluting”-like effect on the main compounds responsible for the lipase inhibition after hydrolysis, as the hydrolyzed extracts had a higher extraction yield (Figure 1) than that of the non-hydrolyzed fenugreek extract at time 0 of hydrolysis (Figure 3). Hence, it seems that those extracts with a lower hydrolysis extraction yield and thus a lower sapogenin content, exhibited the best lipase inhibitory activities, regardless of the temperature of hydrolysis.

Considering these results altogether, it is clearly shown that depending on the MAAH conditions, selective extracts can be obtained for different purposes. Thus, if hydrolyzed extracts with a high sapogenin content were desired to be produced, regardless of the pancreatic lipase inhibitory activity, the optimal MAAH temperature condition would be 140 °C. On the other hand, if hydrolyzed extracts with a very strong lipase inhibitory activity were desired to be produced, regardless of sapogenin content, the optimal MAAH temperatures would be 100 or 120 °C.

Previous information about the impact of the hydrolysis of saponins on the subsequent bioactivity of sapogenin extracts is scarce. We recently demonstrated that the conventional hydrolysis of saponin-rich extracts from quinoa improved their lipase-inhibitory activity, while the bioactivity from those extracts resulting from the hydrolysis of an experimental fenugreek saponin-rich extract was maintained. In such a study, the initial saponin content of the fenugreek extracts (30%) was transformed into extracts with an 8.1% sapogenin content, and still, the lipase inhibitory activity was unaffected [15]. Except for two research articles, we have not been able to find any other study assessing the effect of microwave hydrolysis of saponins on the bioactivity of the resulting products. Colson et al. [32] confirmed that the microwave irradiation of quinoa husk extracts at pH 10 and 150 °C for 5 min led to the most bioactive hydrolysates in terms of their hemolytic/cytotoxic activities, mainly due to the transformation of bidesmosidic (two sugar chains) saponins into monodesmosidic ones (a single sugar chain). In a similar approach, Savarino et al. [49] have quite recently demonstrated that the desulfation of hemolytic saponins extracted from *Holothuria scabra* viscera by microwave hydrolysis at an alkaline pH led to a transformed extract with an absolute lack of hemolytic activity in erythrocytes from bovine blood.

**Figure 6:**
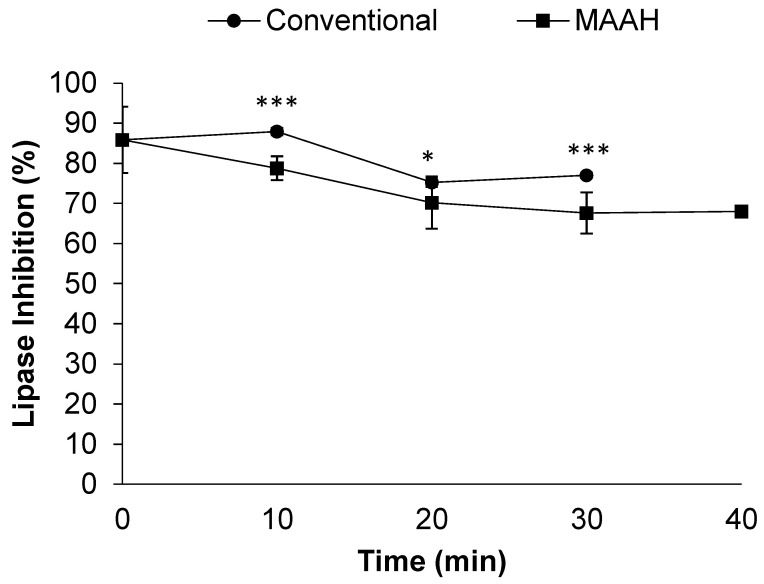
Effect of time on the pancreatic lipase inhibition (%) of fenugreek extracts and sapogenin-rich extracts obtained by conventional hydrolysis and microwave-assisted acid hydrolysis (MAAH). Standard deviations are indicated by error bars. Mean values at the same time of hydrolysis are significantly different if *p* ≤ 0.05 (*) and *p* ≤ 0.001 (***).

Focusing on the effect of time during the MAAH vs. conventional heating, a decrease in the lipase inhibitory activity of the extracts could be observed, along with the time of hydrolysis in both the methods studied (Figure 6). However, a plateau of around 70% of inhibition seemed to be reached at 20 min. Between the two methods studied, hydrolyzed extracts obtained by conventional hydrolysis were slightly more bioactive towards the inhibition of the lipase compared to MAAH (*p* < 0.001), even though the maximum difference between the two methods was barely a 10% inhibition at 10 and 30 min. This could be explained by the higher extraction yield of MAAH compared to conventional hydrolysis, and the subsequent diluting-like effect, as previously mentioned.

No differences were observed in terms of the lipase inhibition between the extracts produced under the three different HCl molarities assayed (Supplementary Materials, Figure S2).

In summary, MAAH allows producing sapogenin-rich extracts that preserve the bioactivity towards the inhibition of the pancreatic lipase enzyme at hydrolysis temperatures lower than 130 °C, but the bioactivity worsens due to higher temperatures and times of hydrolysis, probably related to a dilution-like effect of the inhibitory compounds. Therefore, the bioactivity of these extracts can be modulated depending on the final sapogenin content desired in them, and vice versa.

#### 3.2.2. Effect on the Antioxidant Activity by the DPPH Radical Scavenging Assay

**Figure 7:**
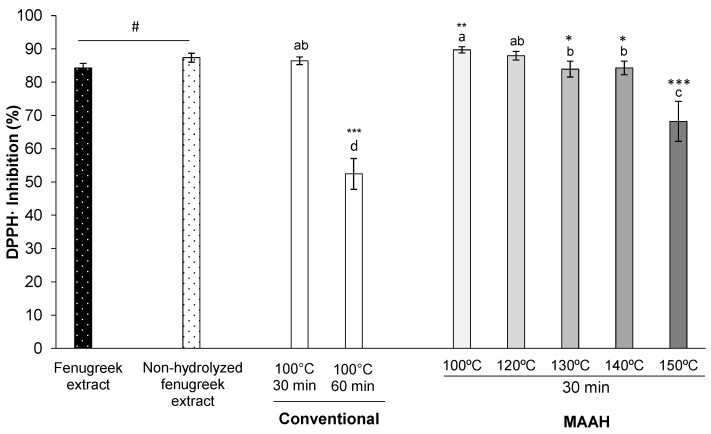
Effect of temperature on the DPPH radical scavenging activity (%) of fenugreek extracts and sapogenin-rich extracts obtained by conventional hydrolysis and microwave-assisted acid hydrolysis (MAAH). Standard deviations are indicated by error bars. Mean values with different letters are significantly different (*p* ≤ 0.05) and mean values are significantly different from the non-hydrolyzed fenugreek extract if *p* ≤ 0.05 (*), *p* ≤ 0.01 (**) and *p* ≤ 0.001 (***). Non-hydrolyzed fenugreek extract is different from fenugreek extract (#, *p* ≤ 0.001). “Fenugreek extract” refers to the commercial saponin mixture as received from the supplier. “Non-hydrolyzed fenugreek extract” refers to the “fenugreek extract” extracted with ethyl acetate at time 0 of hydrolysis.

Concerning the antioxidant potential of the extracts, a very strong radical scavenging activity against DPPH was observed for almost all extracts regardless of temperature or time. We initially assessed the antioxidant activity of both the fenugreek extract and the non-hydrolyzed fenugreek extract (time 0 of hydrolysis) (Figure 7). The antioxidant activity of the two extracts was very high, although that of the non-hydrolyzed one was slightly stronger than the fenugreek extract (87 vs. 84%, *p* = 0.014).

Concerning the effect of temperature during the MAAH (Figure 7), and contrary to what occurred for the lipase inhibition, the antioxidant activity of almost all the hydrolyzed extracts produced under MAAH (100, 120, 130 and 140 °C) was very subtly altered with respect to the reference non-hydrolyzed fenugreek extract and varied in the range of 84–89.7%. Only the hydrolyzed extract produced at 150 °C lost some of its antioxidant activity, 68% (*p* < 0.001). Additionally, it is interesting to remark that the reference condition during conventional hydrolysis (100 °C, 60 min), led to the worst hydrolyzed extract in terms of its antioxidant activity, even worse than MAAH at 150 °C for 30 min.

**Figure 8:**
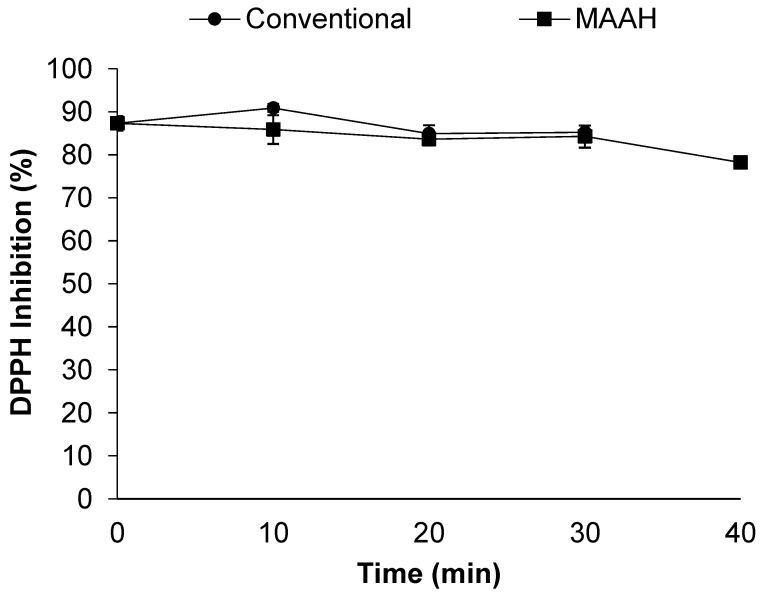
Effect of time on the DPPH radical scavenging activity (%) of fenugreek extracts and sapogenin-rich extracts obtained by conventional hydrolysis and microwave-assisted acid hydrolysis (MAAH). Standard deviations are indicated by error bars.

Concerning the effect of time on the antioxidant activity during the MAAH vs. conventional heating (Figure 8), no differences were observed between the two studied methods at any time studied. Moreover, the antioxidant activity seemed to be quite stable during time and it only noticeably decreased at 40 min of MAAH (78%) compared to time 0 (*p* = 0.01).

No differences were observed in terms of the DPPH inhibition between the extracts produced under the three different HCl molarities assayed (Supplementary Materials, Figure S3).

In short, MAAH allows producing sapogenin-rich extracts from fenugreek that preserve the antioxidant activity observed initially for the raw fenugreek extract, since hydrolyzed extracts are, in general terms, barely affected by temperature or time at the ranges studied.

## 4. Conclusions

MAAH is shown as an adequate alternative method to the conventional acid hydrolysis of saponins for the obtention of sapogenin-rich extracts from fenugreek extracts. Values higher than 120 °C and up to 140 °C are necessary for an efficient MAAH of fenugreek saponins. The sapogenin yield increases during the time of the MAAH reaction, reaching a maximum after 30 min of hydrolysis. Under these conditions of temperature and time of hydrolysis (140 °C, 30 min) the sapogenin yield is superior to that of conventional heating at any time of reaction. Higher MAAH hydrolysis temperatures and times do not improve the hydrolysis extraction yield or the sapogenin content of the resulting extracts but lead to a decrease in both parameters instead. Additionally, the presence of artifacts originating from sapogenins is minor and apparently not temperature or time-related.

MAAH can impact the bioactivity of the hydrolyzed extracts, but the magnitude of such modulation depends on the tested bioactivity and the hydrolysis conditions. Thus, for a preserved inhibitory activity against the pancreatic lipase, moderate MAAH temperatures of up to 120 °C would be preferred. Although it is remarkable that all extracts obtained at the different MAAH conditions show moderate to high bioactivity in all cases. In general, the antioxidant activity of the hydrolyzed extracts is preserved, with all of them exhibiting very strong antioxidant potential. Finally, it is suggested that the magnitude of the multibioactivity of the hydrolyzed extracts obtained by MAAH can be modulated depending on the final sapogenin content desired in them, and vice versa.

Taking all this evidence together, and due to the extraordinarily scarce number of previous studies assessing the hydrolysis of saponins by microwave irradiation and its effect on their further bioactivity, more studies focusing on other sources of saponins may be of interest. This would contribute to the potential of this method as an alternative hydrolysis technique for producing bioactive sapogenin-rich extracts.

## Data Availability

Data is contained within the article or supplementary material.

## Supplementary Materials

The following supporting information can be downloaded at: https://www.mdpi.com/article/10.3390/foods11131934/s1, Figure S1: Sapogenin content (g/100 g) and extraction yield (%) of extracts obtained at different molarities of HCl by microwave hydrolysis at 140 °C for 30 min. Standard deviations are indicated by error bars. Mean values with different letters are significantly different (*p* ≤ 0.05); Figure S2: Lipase inhibition (%) and sapogenin content (g/100 g) of extracts obtained at different molarities of HCl by microwave hydrolysis at 140 °C for 30 min. Standard deviations are indicated by error bars. Mean values with different letters are significantly different (*p* ≤ 0.05); Figure S3: DPPH inhibition (%) and sapogenin content (g/100 g) of extracts obtained at different molarities of HCl by microwave hydrolysis at 140 °C for 30 min. Standard deviations are indicated by error bars. Mean values with different letters are significantly different (*p* ≤ 0.05); Table S1: Sapogenin profile of sapogenin-rich extracts produced from a commercial saponin-rich fenugreek extract under conventional hydrolysis (100 °C, 60 min) or microwave-assisted acid hydrolysis (MAAH, 30 min) at different temperatures (100, 120, 130 and 140 °C); Table S2: Sapogenin profile of sapogenin-rich extracts produced from a commercial saponin-rich fenugreek extract under conventional hydrolysis (100 °C, 60 min) or microwave-assisted acid hydrolysis (MAAH, 140 °C) at different times (10, 20, 30 and 40 min).
