# Supplementary material for: Microwave-Assisted Acid Hydrolysis vs. Conventional Hydrolysis to Produce Sapogenin-Rich Products from Fenugreek Extracts
Source: Foods. 2022 Jun 29;11(13):1934. doi: 10.3390/foods11131934 (PMC9266256; doi:10.3390/foods11131934)
Supplement: Supplementary file 1 [file foods-11-01934-s001.zip › foods-1782157-supplementary.pdf]

Supplementary material

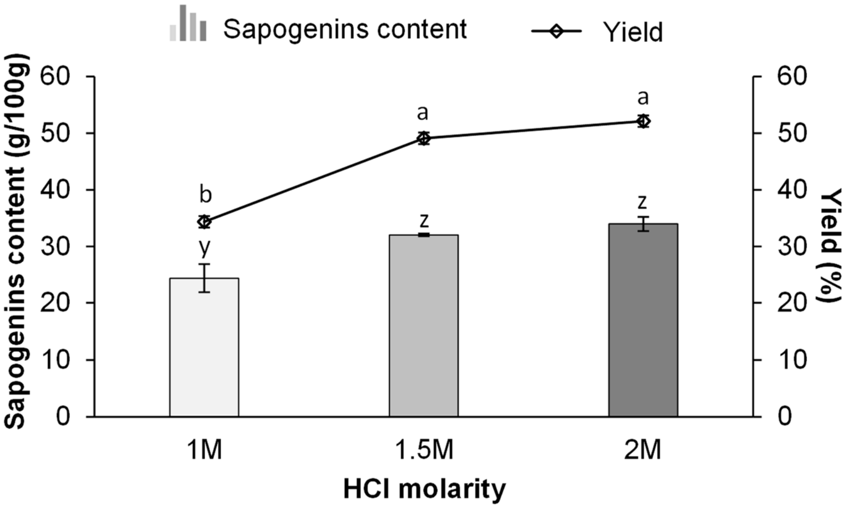

Figure S1. Sapogenin content (g/100 g, n =2) and extraction yield (%, n = 2) of extracts obtained at different molarities of HCl by microwave hydrolysis at 140ºC for 30 min. Standard deviations are indicated by error bars. Mean values with different letters are significantly different ($p \leq 0.05$).

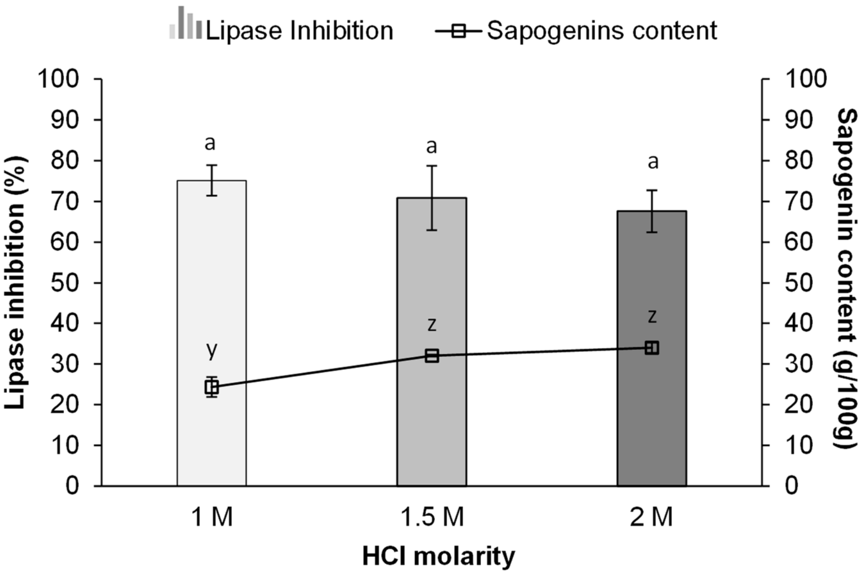

Figure S2. Lipase inhibition (%, n = 2) and sapogenin content (g/100 g, n =2) of extracts at 0.75 mg/mL obtained at different molarities of HCl by microwave hydrolysis at 140ºC for 30 min. Standard deviations are indicated by error bars. Mean values with different letters are significantly different (p ≤ 0.05).

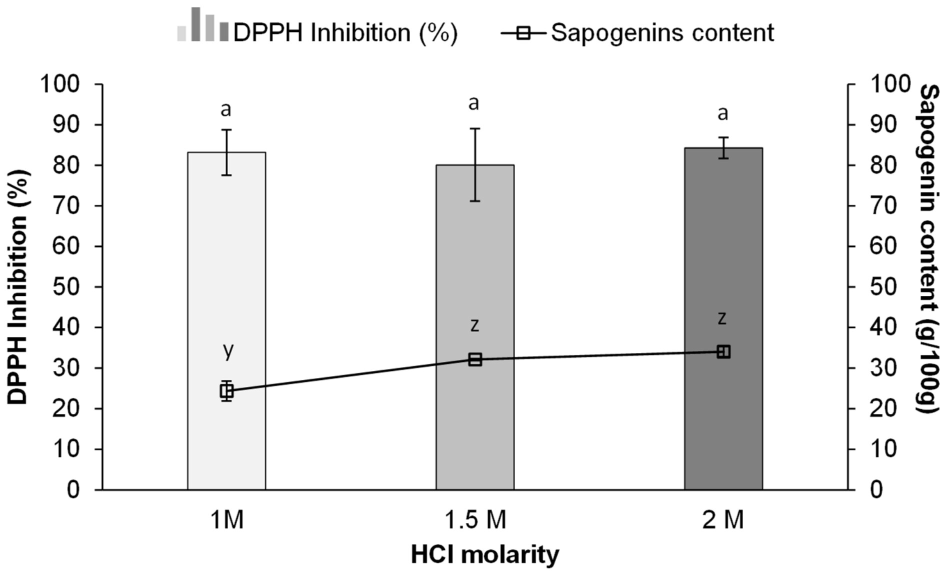

Figure S3. DDPH inhibition (%, n = 2) and sapogenin content (g/100 g, n =2) of extracts at 0.3 mg/mL obtained at different molarities of HCl by microwave hydrolysis at 140ºC for 30 min. Standard deviations are indicated by error bars. Mean values with different letters are significantly different (*p* ≤ 0.05).

Table S1. Sapogenin profile of sapogenin-rich extracts produced from a commercial saponin-rich fenugreek extract under conventional hydrolysis (100 ºC, 60 min) or microwave-assisted acid hydrolysis (MAAH, 30 min) at different temperatures (100, 120, 130 and 140 ºC).

Sapogenin	Conventional Hydrolysis (100 ºC, 60 min)	Microwave-Assisted Acid Hydrolysis (MAAH)				
		100 ºC	120 ºC	130 ºC	140 ºC	150 ºC
**3-5-Spirostadiene derivative**	2.61 ± 0.03	0.61 ± 0.13	0.68 ± 0.10	3.73 ± 0.76	4.38 ± 0.02	2.19 ± 0.18
3-5-Spirostadiene derivative	1.61 ± 0.01	0.52 ± 0.08	0.61 ± 0.05	2.18 ± 0.36	2.52 ± 0.01	1.48 ± 0.10
Steroid sapogenin n.i.	0.48 ± 0.01	0.39 ± 0.00	0.44 ± 0.00	0.51 ± 0.00	0.51 ± 0.02	0.45 ± 0.03
Steroid sapogenin n.i.	0.96 ± 0.01	0.41 ± 0.02	0.44 ± 0.06	1.17 ± 0.02	1.26 ± 0.10	0.95 ± 0.09
Steroid sapogenin n.i.	1.06 ± 0.01	0.41 ± 0.02	0.44 ± 0.03	1.20 ± 0.01	1.27 ± 0.05	0.96 ± 0.01
Diosgenin	9.30 ± 0.06	0.90 ± 0.05	0.94 ± 0.21	11.46 ± 0.10	12.78 ± 0.59	6.87 ± 0.11
Tigogenin	1.59 ± 0.00	0.43 ± 0.02	0.43 ± 0.02	1.94 ± 0.03	2.06 ± 0.04	1.26 ± 0.07
Yamogenin	4.61 ± 0.13	0.72 ± 0.09	0.72 ± 0.07	5.55 ± 0.01	6.24 ± 0.32	3.45 ± 0.01
Neotigogenin	1.76 ± 0.05	0.51 ± 0.06	0.52 ± 0.01	2.11 ± 0.07	2.27 ± 0.12	1.45 ± 0.01
Steroid sapogenin n.i.	0.60 ± 0.02	0.38 ± 0.02	0.38 ± 0.01	0.67 ± 0.02	0.70 ± 0.05	0.52 ± 0.03

Table S2. Sapogenin profile of sapogenin-rich extracts produced from a commercial saponin-rich fenugreek extract under conventional hydrolysis (100 ºC, 60 min) or microwave-assisted acid hydrolysis (MAAH, 140 ºC) at different times (10, 20, 30 and 40 min).

Sapogenin	Conventional Hydrolysis (100 ºC, 60 min)	Microwave-Assisted Acid Hydrolysis (MAAH)			
		10 min	20 min	30 min	40 min
**3-5-Spirostadiene derivative**	2.61 ± 0.03	1.75 ± 0.56	2.73 ± 0.08	4.38 ± 0.02	2.76 ± 0.18
3-5-Spirostadiene derivative	1.61 ± 0.01	1.21 ± 0.31	1.73 ± 0.02	2.52 ± 0.01	1.68 ± 0.08
Steroid sapogenin n.i.	0.48 ± 0.01	0.45 ± 0.02	0.50 ± 0.02	0.51 ± 0.02	0.49 ± 0.00
Steroid sapogenin n.i.	0.96 ± 0.01	0.80 ± 0.16	1.14 ± 0.02	1.26 ± 0.10	1.11 ± 0.00
Steroid sapogenin n.i.	1.06 ± 0.01	0.73 ± 0.13	1.08 ± 0.01	1.27 ± 0.05	1.16 ± 0.05
Diosgenin	9.30 ± 0.06	3.91 ± 1.37	8.67 ± 0.36	12.78 ± 0.59	11.08 ± 1.03
Tigogenin	1.59 ± 0.00	0.79 ± 0.18	1.44 ± 0.03	2.06 ± 0.04	1.79 ± 0.11
Yamogenin	4.61 ± 0.13	2.07 ± 0.64	4.38 ± 0.20	6.24 ± 0.32	5.48 ± 0.49
Neotigogenin	1.76 ± 0.05	0.96 ± 0.22	1.71 ± 0.05	2.27 ± 0.12	2.04 ± 0.13
Steroid sapogenin n.i.	0.60 ± 0.02	0.52 ± 0.04	0.70 ± 0.01	0.70 ± 0.05	0.85 ± 0.04
